# Myxomatosis and Rabbit Haemorrhagic Disease: A 30-Year Study of the Occurrence on Commercial Farms in Spain

**DOI:** 10.3390/ani9100780

**Published:** 2019-10-10

**Authors:** Joan M. Rosell, L. Fernando de la Fuente, Francisco Parra, Kevin P. Dalton, J. Ignacio Badiola Sáiz, Ana Pérez de Rozas, Juan J. Badiola Díez, Daniel Fernández de Luco, Jordi Casal, Natàlia Majó, Jordina Casas, Ricard Garriga, Xosé M. Fernández Magariños

**Affiliations:** 1 Cunivet Service. P.O. Box 518, 43080 Tarragona, Spain; 2 Departamento de Producción Animal, Facultad de Veterinaria, Avda. Profesor Pedro Cármenes s/n, Universidad de León, 24071 León, Spain; f.fuente@unileon.es; 3 Instituto Universitario de Biotecnología de Asturias, Departamento de Bioquímica y Biología Molecular, Universidad de Oviedo, Calle Doctor Fernando Bongera s/n, 33006 Oviedo, Spain; fparra@uniovi.es (F.P.); daltonkevin@uniovi.es (K.P.D.); 4 Institut de Recerca i Tecnologia Agroalimentàries (IRTA)-Centre de Recerca en Sanitat Animal (CReSA), Campus de la Universitat Autónoma de Barcelona, 08193 Cerdanyola del Vallés (Barcelona), Spain; ignacio.badiola@irta.cat (J.I.B.S.); ana.perezderozas@cresa.uab.cat (A.P.d.R.); jordi.casal@uab.cat (J.C.); natalia.majo@uab.cat (N.M.); 5 Departamento de Patología Animal, Facultad de Veterinaria, Universidad de Zaragoza, Calle de Miguel Servet, 177, 50013 Zaragoza, Spain; badiola@unizar.es (J.J.B.D.); luco@posta.unizar.es (D.F.d.L.); 6 Departament de Sanitat i Anatomia Animals, Facultat de Veterinària, Universitat Autónoma de Barcelona, 08193 Cerdanyola del Vallés (Barcelona), Spain; 7 Federació d’Associacions de Cunicultors de Catalunya (FACC). Carrer Ull de Llebre, 13 08734 Olèrdola (Barcelona), Spain; jordinacasas@gmail.com (J.C.); ricard-garriga@hotmail.com (R.G.); 8 NANFOR, Aldea Loureiro, 40, 15980 Padrón (A Coruña), Spain; xm.fernandez@nutreco.com

**Keywords:** animal welfare, disease prevention, myxomatosis prevalence, rabbit haemorrhagic disease incidence

## Abstract

**Simple Summary:**

Myxomatosis has affected the European domestic rabbit in Spain since the end of 1953; and rabbit haemorrhagic disease (RHD) since mid-1988. In this study, we describe the occurrence of myxomatosis; and RHD; on commercial rabbitries visited in Spain; between 1988 and 2018. Annual occurrence of myxomatosis decreased over 30 years. Cases of myxomatosis were detected most frequently between August and March; more cases occurring in September and fewer in June. Two important RHD epidemics occurred; the first in 1988–1989 due to classic RHD virus (RHDV), and the second from 2011 to 2013 due to new calicivirus (RHDVb/RHDV2). Monthly occurrence of RHD in 2011–2018 was higher from April to August. Despite seasonal variations of these diseases; we recommend that young breeders and adults should be protected by vaccination all year round; and on-farm hygiene measures implemented

**Abstract:**

In this retrospective study, we describe the relative occurrence of clinical myxomatosis, and rabbit haemorrhagic disease (RHD), on 1714 commercial farms visited in Spain, between 1988 and 2018. We determined the annual prevalence based on 817 visits to 394 farms affected by myxomatosis. Myxomatosis was more prevalent from August to March, being lowest in June (3%) and highest in September (8.9%). With regard to RHD, we assessed 253 visits to 156 affected farms. We analyzed mean annual and monthly incidence. Two important RHD epidemics occurred; the first in 1988–1989 due to RHDV GI.1 (also known as RHDV), and the second from 2011 to 2013 due to RHDV GI.2 (RHDV2 or RHDVb). These epidemics occurred at times when effective vaccination had not been carried out. Relative monthly incidence in 2011–2018 was higher from April to August (*p* < 0.001). The results we obtained from 1404 necropsies on 102 farms did not clearly relate serosanguinous nasal discharge in rabbits with disease caused by GI.2 infection. We also assessed vaccination schedules used on 200 doe farms visited from the end of 2014 to 2018; 95.5% vaccinated against myxomatosis and 97.5% against RHD. Both diseases remain prevalent; however, effective vaccination has produced a steady decline in myxomatosis and RHDV GI.1 and GI.2 on-farm detection. The maintenance of high hygienic standards will be needed to continue and improve this control. However, further studies are required to investigate the causes of sustained virus presence and vaccine breaks.

## 1. Introduction

The major viral diseases affecting the European domestic rabbit (*Oryctolagus cuniculus*) are myxomatosis and rabbit haemorrhagic disease (RHD) and these have serious effects on the health and welfare of rabbits [1]. The economic perspective is often considerable as an entire rabbitry might be affected. The World Organization for Animal Health (OIE) includes myxomatosis and RHD on its list of notifiable diseases [2]. The etiological agents of both diseases can be transmitted between domestic and wild rabbits through the action of blood sucking/biting insects [3]. The effects of myxomatosis and RHD on the health of wild rabbit populations also have consequences on related ecosystems [4].

Clinical myxomatosis frequently occurs in a subacute or chronic form, with severe productive rhinitis and dyspnea [5,6]. Affected rabbits can have a rapid course to septicemia and death, or have pulmonary lesions, blepharitis, aural, and urogenital swelling, and cutaneous myxomas or, in subacute cases, bacterial superinfection [6,7]. In terms of pathogenicity and virulence, the immune suppressing effect of some myxoma viruses [8] is of particular note. 

RHD is characterized by disseminated necrotic hepatitis [9]; it evolves peracutely or acutely with high mortality. Prior to death, clinical signs derive from severe disseminated intravascular coagulopathy (DIC), with multi-organ failure [10], sometimes with asphyxia, seizures, and intense suffering [11]. There is no treatment; vaccination is the only preventive measure, together with the implementation of biosecurity measures [12]. 

*Myxoma virus* (MYXV) belongs to the *Poxviridae* family and the *Leporipoxvirus* genus. In Spain, myxomatosis was first diagnosed in domestic rabbits towards the end of 1953 [13]. There were outbreaks of classic or typical myxomatosis until 1978, with different degrees of cutaneous clinical expression in the form of pseudotumors: myxomas [7], depending on the susceptibility of the rabbits and viral strains involved [14]. From 1979 onwards, the presence of atypical myxomatosis was described “with decreased cutaneous expression and continued respiratory problems” [7]. Since then, outbreaks of both forms have occurred: classic and atypical or “amyxomatous”, mistakenly referred to as “respiratory” [15]. In a previous study, based on 660 visited farms, we reported a seasonal variation with an increase from October to December [16]. 

Although effective vaccines against myxomatosis have been available for some time the disease persists. “Heterologous” vaccines based on the rabbit (Shope) fibroma virus have been used since 1955 and “homologous” vaccines (e.g., the *SG 33* strain [17], *León 162*, and *VMI 30* strains) since the 1980s with different adjuvants, and administration routes: subcutaneous or intradermic [18,19,20]. 

RHD is caused by rabbit haemorrhagic disease virus (RHDV now termed RHDVG I.1 [21]). In Spain, the first cases of RHD were identified in domestic rabbits in June 1988 [22] and soon after the causative agent was identified by Parra and Prieto [23]. A specific vaccine against this RHDV strain was used in Spain from January 1989, with protection shown to last over one year [24]. During the following years, different RHD genogroups (e.g., GI.1a-d) were identified mainly in other European countries [25,26], but had no serious effects on farms as the available vaccines proved effective (review in [27]). This could explain why the occurrence of RHD in domestic rabbits, was only serious at the start of the 1988–1989 epidemics, coinciding with when the vaccine was not available [11]. Since 1999, a combined vaccine with MYXV and GI.1 virus [28] has been available. Additionally, a bivalent recombinant vaccine of homologous attenuated MYXV, which expresses GI.1 capsid protein (VP60) has been on the market since 2012 [29]. In 2010, a novel RHDV was identified in France [30] and subsequently in Spain: originally RHDV2 or RHDVb now termed RHDV GI.2, affecting rabbits under 35 days old [31] and vaccinated rabbits. The occurrence of disease due to GI.2 in Spain was serious from 2011 until July 2013, when vaccines against it were first used [32]. 

Our aims were to (1) describe the procedures used for diagnosing clinical myxomatosis and RHD on commercial farms between 1988 and 2018, (2) estimate myxomatosis prevalence through farm visits over the 30-year period, (3) calculate the incidence of RHD, and (4), describe MYXV and RHDV vaccination schedules on 200 rabbit doe farms visited from the end of 2014 to 2018. 

## 2. Materials and Methods

Our 30-year study lasted from 11 September 1988, when we visited our first farm affected by RHD, to 11 September 2018. We obtained information from a total of 13,467 visits to 1714 commercial farms. The information gathered was used to generate a dataset with the number of adult breeding rabbits per farm, and rabbit health management. Animal Care and Use Committee approval was not obtained for this study because data were obtained from rabbits raised under commercial conditions, fulfilling European, Spanish and regional recommendations and laws on animal welfare, food safety and environmental protection.

### 2.1. Characteristics of the Farms Visited

**Figure 1:**
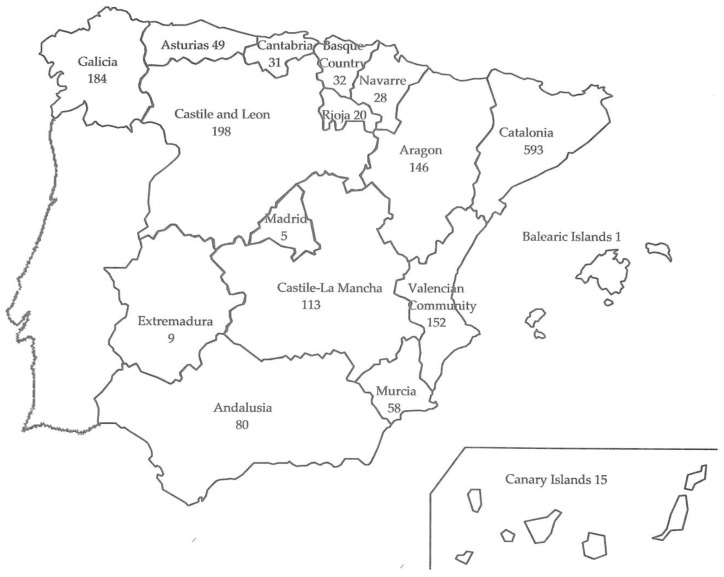
Distribution by Autonomous Communities of 1714 rabbit farms visited in Spain from 11 September 1988 to 11 September 2018.

This retrospective study included all the 1714 farms visited by the first author in Spain. They housed females with or without males or weaned rabbits, only males, or only weaned rabbits. The target populations included all types of rabbits because they were all at risk. Farms in Spain are inscribed in the official database *Registro General de explotaciones ganaderas* (REGA). According to the *Ministerio de Agricultura, Pesca y Alimentación* [33], the number of farms varied greatly throughout the 30-year period, with a marked decrease in those housing >20 does; e.g., over 5000 farms in 1999, whereas in 2017 it had decreased to fewer than 1000. In a preliminary paper [34], we provided a detailed explanation of the farms registered in the REGA database and those visited between 2001 and 2017 (opus cit., Figure 1). At the same time, the average size (number of does) of the farms increased. In addition, we have used a subset of 200 farms housing does, that we have visited since the end of 2014 until the end of 2018, to describe the vaccination programs, among other factors. In our case, a doe was a female that had been serviced once or more. 

### 2.2. Veterinary Visits to the Rabbit Farms

In this study, the clinical information on myxomatosis and RHD was collected by the first author who provided veterinary services to domestic rabbit producers and on-farm observation or examination of the rabbits. All of the farms had been visited by other veterinarians. The visits were due to urgent clinical calls, or to carry out routine check-ups. When classifying urgent visits, we only considered the main cause; myxomatosis and RHD were always priority causes. Nevertheless, cases of zoonotic diseases, e.g., salmonellosis [35], amongst others, were recorded for monitoring and surveillance purposes. Producers were asked about different aspects, such as the number of existing females or males, if any, per farm and the used lines, type of service: mount or artificial insemination (AI), reproduction rhythm and number of batches per barn or farm, amongst others. In addition, we inquired about myxomatosis and RHD vaccination schedules. 

### 2.3. On-Farm Diagnostic Procedures

We observed signs of myxomatosis including prostration or dyspnea, and lesions such as blepharitis and conjunctivitis, ear edema or myxomas. When we detected a suspected case, we checked the anogenital region and palpated the skin for myxomas if they were not visible. These elements, besides the epidemiological features often helped us to issue a presumptive diagnosis. So as to not confound in the on-farm diagnosis, we collected samples for histopathologic study from farms with only some doubtful sick rabbits, for diagnosis of myxoid tissue [36]. Images related to myxomatosis may be seen on [37].

Concerning RHDV GI.1, from 1988 we carried out a campaign of frequent visits, necropsies and sample collection for laboratory analysis, where the O type human blood agglutination test [38] was carried out. When the RHDV GI.2 epidemics occurred in 2011–2013, laboratory diagnosis consisted of RT-PCR and sequencing similar to that described in [31,39] and when it became available with a rapid field analytical test kit (Cer*Test* Biotec, Zaragoza, Spain). We also necropsied adults, young rabbits and weaned rabbits (>35 days old), and carried out histopathologic studies of rabbits under 25 days old. Images related to RHD are available on [40].

### 2.4. Statistical Analysis

The database used contains 13,467 records (farm visits). The variables recorded were occurrence of clinical myxomatosis and RHD (categorical variable) on each farm visit. The relative occurrence was calculated dividing the number of myxomatosis or RHD cases by the total number visits. Following the same procedure as in a preliminary study [16], we only used one visit per farm and calendar month, at most, for these calculations; thus, the database for the analyses comprised a subset with 13,326 visits. Concerning measures of disease occurrence, we followed the criterion of Thrusfield [41]. In the case of clinical myxomatosis, we calculated relative annual and monthly relative prevalence; the reason for this was that we visited farms where the disease could have been an enzootic pattern. In the case of RHD, we analyzed the incidence because in the preliminary assessment of the time between the first visit to each case of RHD and the first day with compatible losses on each farm, the results were as follows: 50.5% of the 109 first-visits during 2011–2018 were made on the first 7 days of the apparent start of each outbreak of RHD.

The analyses were carried out using the SAS statistical package, and the CATMOD, FREQ, or MEAN procedures, depending on the analyses we used [42]. The statistical model (CATMOD procedure) used was the following:

Y_ijk_ = µ + YE_i_ +MO_j_ +e_ijk_ (1)

where Y_ij_ were the dependent categorical variables, occurrence of myxomatosis or RHD on each visit-farm, YE_i_ was the year effect (30: from September 1988 until September 2018), MO_j_ was the month effect (12), and e_ijk_ was the residual effect. We have not analyzed covariates.

## 3. Results

### 3.1. Characteristics of the Visited Farms

Figure 1 shows the number of farms (1714 in total) visited per region in Spain over the 30-year period.

Throughout the study, the farms decreased in number but increased in size (Table 1). The total number of farms visited represented 10-20% of Spanish farms, according to the year. Relative to data in Table 1, with 3-year observation periods, there were in total 190 visits due to myxomatosis and 1618 visits (calendar month), from 11 September 1988 until 11 September 1991.

We visited farms in 47/50 provinces. Most were meat production farms (1704 of the 1714), 4 farms housing Rex rabbits for fur production, 3 with dwarf rabbits and 3 with New Zealand rabbits for laboratory purposes. Most farms housed does (1677 of the 1714), mainly with weaned rabbits in the same farm, and often in separated barns. In addition, there were 14 farms with weaned rabbits only and 23 with males only. On all of the farms visited during the 30 years, the rabbits were kept in conventional individual housing without elevated platforms; however, on the 200 doe farms visited from the end of 2014 to 2018, approximately 77.5 % had installed footrests.

### 3.2. Visits to Farms

We made 13,467 visits to farms during the 30-year study. Our assessment of relative annual and monthly occurrences of clinical diseases was based on one visit per farm and calendar month, at most, as we have explained in Section 2.4 (statistical analysis); hence the figure 13,326. In total, 862 visits were made due to myxomatosis, 817 without repeated visits in the same month (–5.2%), to 394 farms. For RHD, 344 visits were made, 253 also without monthly repetitions (–26.5%), to 156 farms. Table 2 shows the number of visits for all the months. 

The number of monthly visits during the 30 years was similar; this contributed to preventing bias.

### 3.3. Diagnosing Myxomatosis

We based our on-farm diagnostic procedures of myxomatosis on clinical examination of live rabbits, and sometimes with dead animals. Differential diagnosis was necessary (1) in 2-week-old rabbits, (2) in cases of concurrent diseases such as dermatophytosis or rhinitis, (3) in peracute forms resulting in death, and (4) in rabbits previously vaccinated against myxomatosis. An atypical case of myxomatosis in a female can be seen in a video on our website: https://www.cunivetservice.com/en [43].

### 3.4. Diagnosing Rabbit Haemorrhagic Disease

During the 344 visits and in necropsies we made due to RHD, we observed different degrees of evolution of the disease based on the contents of the stomach (full or empty). We have related a full stomach with an acute process; according to Cooke [44]: “(sick) rabbits…may even continue to eat sporadically a few hours or sometimes minutes before death”. During the first years of the study, we found affected adults and weaned rabbits (>35 days old), with few 23–25-day old rabbits being affected, as observed in a previous study [20]. In January, 2011 we observed a particularly interesting case of affected young rabbits (25-days old) due to RHDV GI.1. Months later, we started to diagnose disease due to RHDV GI.2 infection. During the 2011–2013 GI.2 epidemics, vaccines produced with classic GI.1 strains were not effective and the number of calls to farms increased. Unlike during the previous GI.1 epidemics (1988–2010), during the GI.2 outbreaks we necropsied adults [45] and young rabbits (10–35 days) or weaned rabbits. We also examined samples from on-farm suspected but somewhat unclear cases and several specimens were sent to the laboratory, some of which were confirmed to be bacterial septicemia, or bacterial pneumonia and RHD, simultaneously. From 2011 onwards we decided to take serosanguinous nasal discharge into consideration. Between July 2011 and September 2018 we performed 1404 necropsies on 102 farms, the apparent cause of death being RHD caused by GI.2. We observed serosanguinous nasal discharge in 18.6 % of cases (261/1404).

### 3.5. Analysis of the Studied Risk Factors

Table 3 shows the results of the analysis of variance for the categorical data, corresponding to the model explaining the occurrence of myxomatosis and RHD, showing the significance of the year and month effect. 

### 3.6. Relative Prevalence of Myxomatosis

#### 3.6.1. Annual Prevalence of Myxomatosis

**Figure 2:**
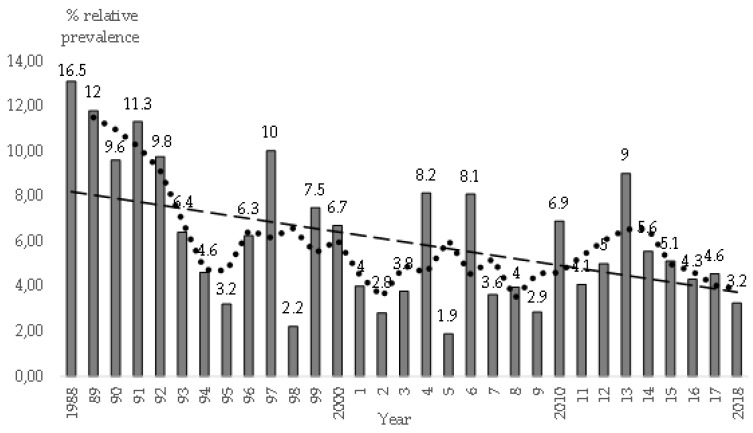
Mean annual prevalence of visited farms with clinical myxomatosis, rolling average for three years (dotted line) and trend, based on 817 visits to 394 affected farms. A total of 13,326 visits (calendar month) were made to 1714 farms in Spain, from 1988 to 2018.

Figure 2 shows mean and annual (relative) prevalence of myxomatosis. We have also included the rolling average and trend. For example, there were 32 visits due to myxomatosis and 194 total (calendar month) visits from 11 September 1988 until 31 December 1988 (16.5% annual relative prevalence).

Mean relative annual prevalence of clinical myxomatosis on the farms was 6.5% throughout the 30-year study period. There were apparently three prevalence peaks (rolling average): 1997, 2005, and 2013; we did not analyze the causes, e.g., the climate variables. Annual prevalence decreased from 12.6% in 1989 to 5.3% in 1995 (rolling average). Our hypothesis is that vaccination schedules were improved. Vaccines made with attenuated homologous virus strains were not used extensively until the 1990’s. Thus, on 95.5% of 200 farms housing does visited from the end of 2014 to 2018, future breeders and adult females were vaccinated against myxomatosis; “homologous” vaccines were used on 90.6% of them. We paid particular attention differentiating to which groups of does the booster should be applied; for example, taking into account variables such as a) the type of farm, e.g., in selection farms, the does were not revaccinated, (b) we asked when females were last vaccinated with “homologous” vaccine (in general, we recommend revaccination from 6 to 8 months onwards). Furthermore, we considered cases where the maternal or the vaccinal immunity might have waned or the immune system was affected, due to predisposing risk factors such as gastroenteric diseases, or enabling risk factors, e.g., overexposure to vaccine antigens. In the case of predisposing risks, we recommended revaccination, but in other situations (e.g., overexposure) we did not suggest revaccination.

#### 3.6.2. Monthly Prevalence of Myxomatosis

**Figure 3:**
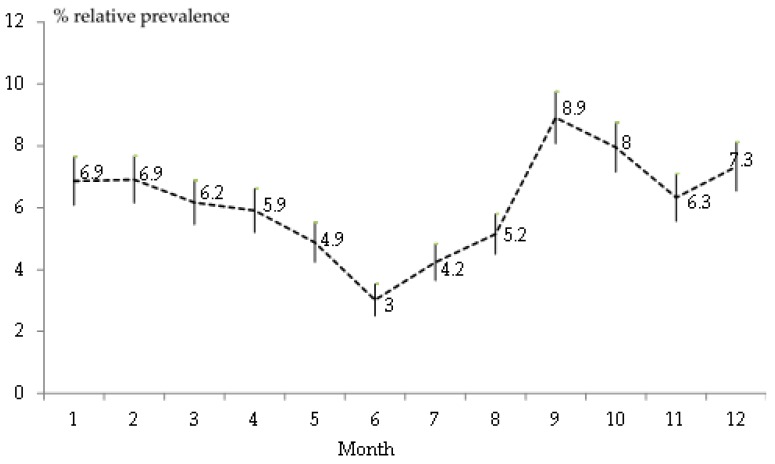
Monthly relative prevalence of clinical myxomatosis and standard error of the mean, based on 817 visits to 394 affected farms. A total of 13,326 visits (calendar month) were made to 1714 farms in Spain, from 1988 to 2018.

We analyzed the seasonal effect based on results for relative monthly prevalence. For instance, from Table 2, dividing 71 visits due to myxomatosis during January of the 30-year, by 1035 total visits (calendar month) (Figure 3). Mean monthly relative prevalence was 6.1%. We observed the highest peak in September (8.9%) and the lowest prevalence in June (3%).

In Spain, mosquitoes and flies are usually more abundant on farms from August to October, although this varies from farm to farm; in the present study we did not find flea infestations. 

### 3.7. Relative Incidence of Rabbit Haemorrhagic Disease

#### 3.7.1. Annual Incidence of RHD

**Figure 4:**
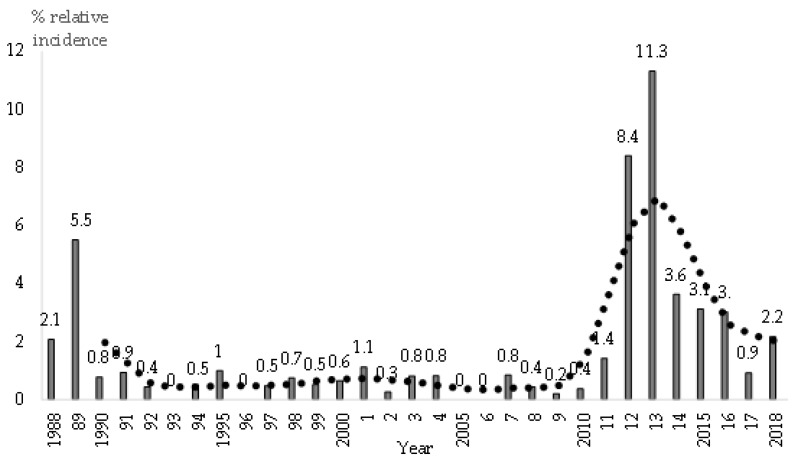
Relative annual incidence of rabbit haemorrhagic disease (RHD), based on 253 visits to 156 farms, and rolling average for 3 years (dotted line). A total of 13,326 visits (calendar month) were made to 1714 farms in Spain, from 1988 to 2018.

Figure 4 shows the evolution of the relative annual incidence of RHD. The annual mean throughout the 30-year study period was 1.9%.

There were two serious epidemics, during 1988–1989 and 2011–2013, with different mean relative incidences, reaching maximum levels of 5.5% in 1989 and 11.3% in 2013. The GI.1 epidemic occurred due to lack of protection as there was no vaccine available; while the GI.2 epidemic occurred demonstrating a lack of cross protection of the GI.1 based vaccines. With regard to vaccination against RHD, on the 200 doe farms visited from the end of 2014 to 2018, 97.5% of the does were systematically vaccinated against RHDV GI.2 or against RHDV GI.1 as well. In June 2019, GI.1 or GI.2 based vaccines were available. In our vaccination schedule against RHD, following the criterion of Capucci et al. [46], we suggested vaccinating future breeders against *classic* RHDV (GI.1) and against new RHDV (GI.2); boosters were only recommended against GI.2 in adults. Following the serious 2011–2013 epidemics, the incidence of RHD remained higher than during 1990-2010. In our opinion, this was due to the recurrent outbreaks of RHD on the same farm, mainly in non-vaccinated weaned rabbits or runts. 

#### 3.7.2. Monthly Incidence of RHD

**Figure 5:**
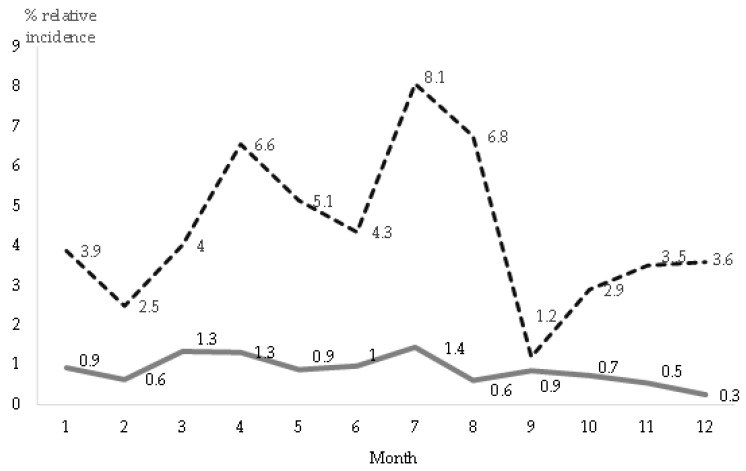
Relative monthly incidence of RHD during 1988-2010 (continuous line), due to RHD virus (RHDV) GI.1, and during 2011–2018 (dotted line), due to RHDV GI.2, based on 253 visits to 156 affected farms. A total of 13,326 visits (calendar month) were made to 1714 farms in Spain.

The evolution of relative monthly incidence is shown in Figure 5, for each of the two studied periods and compatible with two types of RHD due to different lagoviruses (GI.1 and GI.2). Season was a risk factor enabling RHD. From 2011 onwards, RHD incidence was higher (*p* < 0.001) during spring and summer (from April to August), than in autumn and winter (from September to March).

There were no significant differences in relative monthly incidence during the first period (1988–2018), probably due to the lower incidence of RHD. 

## 4. Discussion

In relation to the limitations of this retrospective study, and the characteristics of the visited farms, although this was a convenience sample, as the first author had access to the farms; the total number in the study, 1714, was high. Besides the described sizes of the visited farms, there were several risk factors related with housing and management practices, not recorded for all the farms; e.g., the number of batches per farm, the line of rabbits, the rhythm of reproduction, and so on. We did not analyze these factors to avoid confounding or bias. In a previous study we described several of these variables in detail [34]. Concerning myxomatosis, the all-in all-out system allowed cleaning and disinfection of the barn after the sale of the rabbits, which had sanitary advantages, e.g., avoiding contagions (results not presented), in agreement with Huneau-Salaün et al. [47]. 

The aim of diagnosing myxomatosis on farms was at herd level, not clinical-individual assessment per se, which would be of interest in pet rabbits [48], but early detection and culling of sick rabbits, as a key biosecurity measure. On-farm differential diagnosis was necessary, e.g., on farms housing vaccinated rabbits; in such cases, signs and lesions could be moderate, often with a mild clinical aspect. We also had difficulty with the clinical interpretation of some cases of unapparent carriers of MYXV being detected after vaccination, as indicated in [49]. 

In relation to RHD diagnosis, we did not score the clinical events in all of the affected rabbits, which would be more recommendable when examining smaller colonies [50]. From mid-2011 onwards we paid special attention to serosanguinous nasal discharge, which Liu et al. [51], described as typical of RHD caused by the classic virus (RHDV GI.1). This finding is of interest for two reasons. Firstly, because according to our observations, serosanguinous nasal discharge is not a pathognomonic sign of RHD caused by GI.2; we coincide with [50]. Evidently, the most serious fact is that rabbit producers rule out the presence of RHD when serosanguinous nasal discharge is not observed. Secondly, we observed the presence of blood on the nose in cases of myxomatosis, rhinitis and pneumonia, acute mastitis, in rabbits affected by mucoid enteropathy (similar to epizootic rabbit enteropathy) [52]; this could also have occurred due to the existence of *Clostridium* spp., which produces hemolytic toxin [53].

This 30-year study provided epidemiological information of myxomatosis and has enabled us to better assist the rabbit producers with their vaccination’s schedules. Also, as reported in a previous study it has made the continuous training and motivation of farm staff possible, based on technical evidence [34]. Vaccination is a key aspect of myxomatosis control. According to our observations, the percentage of farms using vaccines elaborated with homologous strains of MYXV increased from 1988 to 2018; this could have a favorable effect on decreasing the prevalence of myxomatosis. During 2014–2018 producers vaccinated against myxomatosis on 95.5% of the 200 visited farms; 90% of vaccines used were “homologous”. From 2010 till 2013 there were 274 visited rabbitries: 93.6% vaccinated, 88% with a “homologous” one. In 1994 we visited 193 farms; producers vaccinated against myxomatosis in 81% of cases. In the same year, 59% used “homologous” vaccines either alone or after administering a “heterologous” one [32]. We explicitly monitored and supervised the occurrence of myxomatosis, the protection of vaccines on farms, and the diagnosis of side effects, as indicated by Knight-Jones et al. [54]. Post-vaccination side effects on visited farms could not be related to each individual, as is the case in pet rabbit clinics [55], nor was it possible to study each side effect in detail. Possible vaccine failures due to causes related to its origin, transport or inadequate administration, is an interesting matter (as pointed out in [56]). Apart from errors related to biosecurity, e.g., inefficient culling of sick animals showing mild signs, such failures could also explain the endemic persistence of myxomatosis, even though vaccination was implemented on 95.5% of the farms we visited.

Our monthly visits from 1988 to 2018 were homogeneous; therefore, it was possible to compare the relative monthly prevalence of clinical myxomatosis. In relation to epidemics between November and March, when temperatures were low, Fenner and Ratcliffe [14] described the favorable effect of cold weather, as MYXV is thermolabile; “the disease is less severe and lethal at higher temperatures”. However, “”ambient temperature has no direct effect on the course of RHD in infected rabbits” [44]. In winter, MYXV causing atypical myxomatosis is airborne, whether insects are present or not; however, evidently, they contribute to the spread of the disease in summer and autumn. We advised producers to heat their doe barns to prevent cold weather from weakening their immune system, in accordance with Moberg [57]. Our results suggest that myxomatosis occurrence in domestic rabbits is similar to that observed in wild rabbits by Ross et al., [58] during 1971–1978. Arthur and Louzis [59] studied classic myxomatosis during 1953–1965, and the atypical form since 1979; in the first case, there were more epidemics in August–October, whereas in the second, they also occurred in winter. In Spain, according to Villafuerte et al [60] from 2003 to 2009 there was a higher occurrence of myxomatosis in wild rabbits during summer and autumn. Some years, there were outbreaks during winter [61], depending on the rainfall and the availability of food, the reproductive activity and the presence of susceptible young rabbits [62].

Clinical myxomatosis was clearly endemic on several farms; we could not exclude the cases of unapparent carriers [63], the epidemiological importance of which was difficult to determine, as in the case of RHD, according to [64]. For this reason, and despite seasonality, we recommend keeping future breeders and adult rabbits systematically protected by vaccination, as we have already suggested [15]. We do not recommend “heterologous” vaccines in pregnant does, in agreement with [49]. We diagnosed fibromatosis on a number of occasions, in spontaneous cases in adults, as indicated in [65] or compatible with vaccination in young rabbits under 25–28 days old [5]. Our interest also lies in concurrent diseases, such as digestive and respiratory diseases, previously studied by Marlier et al. [66], taking into account the immune suppressing effect of MYXV [67], and their interactions. With regard to immunoprophylaxis, we wonder how so many cases of myxomatosis occur despite the high percentage of farms applying vaccination. Greater knowledge of the protection of breeding rabbits and application of boosters is a key challenge for the future. In addition, continuous progress must be made in biosecurity practices.

In reference to RHD, we do not know the causes of higher incidence during April–August. Perhaps this epidemiological result differs from the wild, due to climate and environmental variables affecting wild rabbits more intensively [68]. Producers used the vaccines when they were available; in the present study the ratio was 97.5%. On 274 farms visited during 2010–2013, 95% of the does were vaccinated against GI.1; vaccine made with GI.2 virus was first used in Spain in July 2013 [32]. We observed outbreaks of RHD in different farms, and recurrently on same farms. Besides the susceptibility of the host, this could have been due to the persistence of the virus on the farm. Henning et al. [69] observed that the virus remained infective for up to three months under field environment conditions. Another possibility was the presence of the viruses within the area around the farm; e.g., due to the existence of carrier micromammals [70]. The possibility of asymptomatic infections with carriers [71] and excreters existing for 2 months after vaccination is of great interest in the control of RHDV GI.2 [50]. 

## 5. Conclusions

In this retrospective study we assessed the relative occurrence of myxomatosis and rabbit haemorrhagic disease (RHD) in domestic rabbits. To do this, we used a database containing information on 13,326 visits to 1714 farms, located in Spain. The study lasted from 1988 to 2018. Our data indicate two RHDV epidemics: 1988–1989 due to RHDV GI.1, and 2011-2013 due to RHDV GI.2. The data show that the GI.1 epidemics occurred during periods of lack of protection. In 1988–1989 there was no vaccine available. While the GI.2 epidemic occurred demonstrating a lack of cross protection of the GI.1 based vaccines. Agreeing to our observations, serosanguinous nasal discharge is not linked to RHD caused by RHDV GI.2. Analysis of annual and monthly relative prevalence of myxomatosis and mean relative incidence of RHD according to year and month suggest that these variables affect both diseases. Therefore, it may be inferred from our study that future breeders and adults should be systematically protected by vaccination, particularly before the periods when occurrence is higher. Both diseases remain prevalent; however, effective vaccination has produced a steady decline in myxomatosis and RHDV GI.1 and GI.2 on-farm detection. The maintenance of high hygienic standards will be required to continue and improve this control. However, further studies must be carried out to investigate the causes of sustained virus presence and vaccine breaks. 

## Figures and Tables

**Table 1:** Visits for periods of three years due to myxomatosis (myxo.) and rabbit haemorrhagic disease (RHD), and mean sizes (number of does) of farms visited from 1988 to 2018.

Annual Periods ^a^	N Visits (^c^)	N Farms	Mean Size	N Visits due to myxo. (^c^)	N Farms with myxo.	N Visits due to RHD (^c^)	N Farms with RHD
1988–1991	1633 (1618)	470	363	195 (190)	105	55 (45)	39
1991–1994	1256 (1252)	433	381	101 (97)	68	5 (5)	4
1994–1997	1292 (1291)	468	447	82 (81)	70	6 (6)	6
1997–2000	1107 (1104)	327	585	60 (57)	30	8 (8)	6
2000–2003	1231 (1231)	255	773	46 (46)	34	6 (6)	6
2003–2006	1065 (1058)	313	859	65 (58)	45	6 (6)	6
2006–2009	1269 (1268)	316	947	50 (49)	36	6 (6)	6
2009–2012	1818 (1806)	307	982	92 (88)	57	76 (58)	41
2012–2015	1465 (1401)	242	926	104 (92)	43	137 (85)	55
2015–2018	1331 (1312)	196	985	67 (59)	33	39 (28)	15
Total 1988–2018	13,467 (13,326 ^c^)	1714 ^b^	-	862 (817 ^c^)	394 ^b^	344 (253 ^c^)	156 ^b^

^a^ The annual periods of time were from 11 September 1988 to 11 September 1991 and from 12 September 1991 to 11 September 1994, and so on. ^b^ These were different farms, not the sum of farms. ^c^ In brackets: visits for calendar month used in the studies on occurrence.

**Table 2:** Visits per month due to myxomatosis (myxo.) and rabbit haemorrhagic disease (RHD), and number of farms visited from September 1988 to September 2018.

Month ^a^	N Visits Total (^b^)	N Visits due to myxo. (^b^)	N Farms with myxo.	N Visits due to RHD. (^b^)	N Farms with RHD
January	1054 (1035)	89 (71)	71	20 (17)	17
February	1122 (1113)	81 (77)	76	18 (13)	13
March	1176 (1165)	80 (72)	71	31 (26)	25
April	1113 (1098)	66 (65)	63	44 (32)	30
May	1131 (1126)	56 (55)	52	26 (22)	22
June	1107 (1091)	35 (33)	32	37 (23)	20
July	1195 (1180)	50 (49)	49	54 (42)	32
August	1157 (1145)	60 (59)	57	39 (26)	23
September	1160 (1155)	104 (103)	99	12 (10)	10
October	1151 (1131)	97 (90)	83	27 (16)	16
November	1001 (995)	64 (63)	62	19 (13)	13
December	1100 (1092)	80 (80)	76	17 (13)	12
Total	13,467 (13,326 ^b^)	862 (817 ^b^)	394 ^c^	344 (253 ^b^)	156 ^c^

^a^ The total number of visits for all the months of January, February, and so on, during the 30-year study. ^b^ In brackets: visits for calendar month. ^c^ These were different farms, not the sum of farms.

**Table 3:** The CATMOD (analysis of categorical model) of the risk factors year and month for the occurrence of myxomatosis and rabbit haemorrhagic disease (RHD), from 1988 until 2018.

Source	DF	Myxomatosis		RHD	
		Chi-Square	Pr > Chi-Sq	Chi-Square	Pr > Chi-Sq
Year	29	178.61	<0.0001	226.13	<0.0001
Month	11	44.87	<0.0001	36.65	<0.0001
